# Assessment of the Psycho-Emotional State Induced by Open-Skill Sport Activity: An Electroencephalography-Based Study

**DOI:** 10.3390/s26041198

**Published:** 2026-02-12

**Authors:** Erica Iammarino, Ilaria Marcantoni, Sebastiano Grillo, Laura Burattini

**Affiliations:** Department of Information Engineering, Engineering Faculty, Università Politecnica delle Marche, 60131 Ancona, Italy; e.iammarino@pm.univpm.it (E.I.); i.marcantoni@staff.univpm.it (I.M.); s1129841@studenti.univpm.it (S.G.)

**Keywords:** mental involvement, electroencephalogram, brain rhythms, sport

## Abstract

Electroencephalography (EEG) is an effective tool for monitoring the psycho-emotional state induced by open-skill sport activities characterized by dynamic environments and unpredictable situations, offering objective insights into mental engagement. This study aims to characterize the psycho-emotional state induced by table tennis sport activity by exploiting EEG-derived biomarkers. The ‘Real World Table Tennis’ database was analyzed, which includes EEG signals of 25 subjects acquired before, during and after playing table tennis. After preprocessing, 30-s EEG epochs were recursively extracted every 5 s. For each epoch, EEG rhythms were extracted and combined to obtain 37 engagement indexes, defined as ratios of two or more EEG rhythm powers. Median trends of each index were obtained for five cortical regions, and the Wilcoxon signed-rank test was applied to assess significant temporal changes. Results show that engagement indexes can effectively characterize psycho-emotional dynamics during table tennis, capturing the transition from resting to game phase in all cortical regions and exhibiting an increasing trend when having beta/alpha in their definition, and a decreasing trend when having high-frequency rhythms in the denominator. Our findings demonstrate the feasibility of using engagement indexes to monitor psycho-emotional states induced by open-skill sports and provide a framework for investigating mental engagement over time.

## 1. Introduction

Psychological and emotional factors are increasingly recognized as crucial components of athletic performance, complementing traditional physical conditioning. Factors such as selective attention, mental load, stress management, decision-making readiness, and the capacity to maintain focus under pressure are key determinants of sport performance and are estimated to account for approximately 40–90% of performance outcomes [1,2]. Despite this recognition, there are still gaps in the objective measurement and applicability of these psycho-emotional factors, as most available assessment methods rely on post-competition tests or self-assessment questionnaires that cannot objectively capture and monitor in real time the athletes’ cognitive and psycho-emotional states induced by sport activity [3,4].

The electroencephalogram (EEG) has emerged as a promising method to overcome these limitations, attracting growing interest in this context. By capturing the electrical activity of the brain with millisecond temporal resolution, the EEG allows for tracking cognitive and emotional processes as they unfold during real-world scenarios and for mapping both the location and the timing of the underlying neural activity. According to the International Federation of Clinical Neurophysiology, brain oscillations are traditionally classified into distinct frequency bands, defined as delta (<4 Hz), theta (4–8 Hz), alpha (8–13 Hz), beta (13–30 Hz), and gamma (>30 Hz). The spectral power of these oscillations has been associated with characteristic mental states and cognitive processes [5]. More specifically, increased alpha power is typically observed during relaxed and wakeful states, beta power is associated with heightened attention, delta and theta power have been linked to reduced alertness and memory encoding and retrieval, respectively, and gamma power supports complex information processing and integration of task-relevant stimuli, with gamma–theta interactions also being related to the processing of attended stimuli [5,6,7]. Beyond the interpretation of single EEG frequency bands, which may not be all-inclusive markers, a more comprehensive characterization of mental involvement can be achieved through composite measures known as engagement (or involvement) indexes [8]. As reported in a recent systematic review [9], these indexes are computed as ratios between the spectral power of different brain rhythms, allowing a more comprehensive assessment of psycho-emotional and cognitive states.

The relevance of EEG-based monitoring is particularly pronounced in open-skill disciplines, which are characterized by dynamic environments, rapid movements, and unpredictable situations [10]. In these circumstances, it is particularly important to monitor athletes’ ability to regulate stress (e.g., distinguishing between adaptive and maladaptive stress), as well as the risk of psychological fatigue or burnout. Among open-skill sport activities, table tennis stands out for its high cognitive intensity, extreme speed of play, simultaneous involvement of the entire body, and constant need to process and integrate visuomotor feedback. This makes real-time monitoring particularly valuable, as it allows these cognitive variations to be captured precisely when they occur. Real-time observation of the mental states induced by sports activity not only provides opportunities for scientific investigation but also carries important practical implications for promoting athletes’ psychophysical well-being. EEG band-specific oscillatory power can capture and reflect dynamic functional regulation and is modulated according to processing demands. These changes are continuously functionally tuned by task demands, behavioral goals, and internal states. As a consequence, band-power-based EEG biomarkers, such as engagement indexes, seem to be the most appropriate to follow the rapid psycho-emotional demands involved in open-skill sports [11,12]. Sport-induced changes in engagement indexes, as detected through EEG recordings, may represent objective biomarkers that complement traditional performance metrics, paving the way for the development of personalized cognitive strategies that can be applied in sports, training, or directly in competition. In this scenario, EEG-based techniques such as neurofeedback and biofeedback are becoming increasingly popular, as they are capable of providing athletes with immediate feedback on brain activity or specific physiological parameters, helping to enhance self-regulation skills and to improve concentration, emotional control, and stress resilience [2,13].

In light of these premises, this study aims to characterize the psycho-emotional states induced by open-skill sport activity by exploiting EEG-derived biomarkers. More specifically, the analysis will examine the temporal trend of mental engagement indexes during a session of table tennis in various cortical regions in order to assess whether and how they reflect brain adaption to the different cognitive demands that characterize the sport context.

## 2. Related Works

To provide a comprehensive overview of the current state of the art in EEG-based approaches for characterizing the psycho-emotional states induced by sport, a literature review was conducted in Scopus in July 2025. The literature search strategy was designed to cover three areas of interest: (1) the use of EEG as a method for assessing brain activity, (2) the psycho-emotional state as the object of assessment, and (3) sport or physical activities as the field of application. Only English-language articles published within the past 10 years were considered. The articles resulting from the automatic literature search were then manually screened in order to exclude studies in which EEG analysis was absent or only mentioned but not addressed, the study population was not healthy, and/or the research was not related to sport or physical activities. After screening, a total of 16 articles were included [14,15,16,17,18,19,20,21,22,23,24,25,26,27,28,29].

The reviewed literature covered a wide range of sport disciplines. Shooting was the most frequently investigated, followed by table tennis and karate, reflecting a predominance of open-skill and precision sports; other examined disciplines were baseball, soccer, athletics, hockey, and skiing. Among the studies focusing on open-skill sports, only Studnicki and colleagues made the dataset publicly available [14]. Studies also varied in their acquisition protocols, with EEG signals recorded mostly using wearable devices in athletes while practicing sport in real-world or in simulated scenarios (e.g., virtual reality), immediately before or after practicing the sport activity, under resting-state conditions or while performing specific cognitive tasks. Notably, resting-state EEG measurements were included in half of the articles, enabling the investigation of baseline neural activity and/or recovery-related dynamics. However, none of the reviewed studies exploited these measurements to examine how mental engagement changes over time based on the different cognitive demands induced by sport.

Spectral analysis emerged as the predominant methodological approach, having been employed in 14 out of 16 studies, with power spectral density (PSD) typically estimated through fast Fourier transform (FFT)-based methods on Hanning-windowed segments or through Welch’s method. Among EEG rhythms, the most frequently studied to characterize psycho-emotional or performance-related states were theta, alpha, and beta activity. In four of these studies, in addition to rhythm powers, some ratio indexes, specifically beta/alpha, theta/beta, theta/alpha, and beta/(alpha + theta), were computed as markers of cognitive engagement, stress, or emotional regulation [16,22,25,26]. Connectivity analyses were less common, appearing in five studies (31%) and encompassing measures such as coherence, phase lag index, and weighted phase lag index to quantify functional interactions between cortical regions. Analyses predominantly targeted frontal regions, reflecting their central role in attention and emotional regulation, while parietal and occipital areas were considered especially in alpha-related investigations. Furthermore, more than half of the included works were published after 2021, indicating a growing interest in EEG-derived biomarkers for understanding psycho-emotional states in sports contexts.

## 3. Materials and Methods

### 3.1. Description of the Dataset

The population analyzed in this study comes from the “Real World Table Tennis” database created by Studnicki and colleagues and made publicly available on OpenNeuro (https://doi.org/10.18112/openneuro.ds004505.v1.0.4, accessed on 28 April 2025). The database includes data from 25 healthy participants (15 males and 10 females) with an average age of 21.9 years. All participants were right-hand dominant, had normal or corrected-to-normal vision, reported no musculoskeletal or neurological injuries, and had a wide range of table tennis and racquet sport experience. Written informed consent was obtained from all individuals enrolled in the study [30,31].

**Figure 1:**
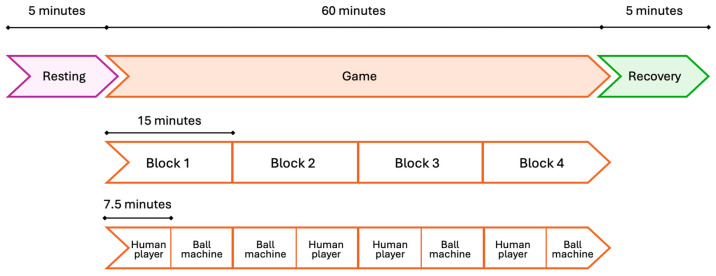
Schematic representation of the phases of the acquisition protocol used [27].

The database contains high-density EEG data, together with neck electromyography and inertial measurement unit (IMU) acceleration data, acquired while participants played real-world table tennis. More specifically, the acquisition protocol consisted of an initial 5 min standing baseline (resting phase), a 60 min session of table tennis playing with a ball machine and a human player (game phase), and a final 5 min standing baseline (recovery phase). The game phase was divided into four 15 min blocks, as shown in Figure 1. Within a single block, participants played a continuous 7.5 min trial with a human player (either cooperatively or competitively) and three 2.5 min trials with a ball machine [31]. The EEG data was recorded from a custom-made dual-layer EEG system (BrainVision ActiCAP snap sensors, BrainVision LLC., Morrisville, NC, USA) designed to mitigate motion-related artifacts, consisting of 120 scalp electrodes referenced to electrode CPz. Each scalp electrode was mechanically coupled to a noise electrode electrically isolated from the scalp, so that any sudden electrode movement relative to the head was captured by both layers and regressed out from the neural signal before further preprocessing. The scalp electrodes were arranged according to the extended 10–20 international system and consisted of active Ag/AgCl sensors. Data were acquired using BrainVision LiveAmp 64 amplifiers (BrainVision LLC., Morrisville, NC, USA) with a 24-bit resolution and an input noise below 2 µVpp. The sampling frequency was 500 Hz, while electrode impedance was kept below 20 kΩ. IMUs were placed on the paddles, ball machine, table, and net to record acceleration peaks that served as precise event markers for the EEG data. Indeed, IMUs and EEG signals were synchronized, and EEG signals were marked according to the timing of the protocol phases and of relevant events occurred during the game phase. Moreover, EEG data were already resampled to 250 Hz and high-pass filtered at 1 Hz using a zero-phase finite impulse response (FIR) filter [31].

### 3.2. Preprocessing and Feature Extraction

EEG data were analyzed using Matlab R2024b and EEGLAB toolbox (version 2025.0.0). Preprocessing included average re-referencing, low-pass filtering using a zero-phase non-causal Hamming windowed sinc FIR filter with a cut-off frequency equal to 80 Hz, powerline noise removal using CleanLine plugin, and artifact removal using independent component analysis (ICA). More specifically, ICA was performed using the Extended Infomax algorithm, and no dimensionality reduction via principal component analysis was applied. Independent components (ICs) were labeled using the ICLabel plugin [32], an automatic machine learning-based classifier that assigns each IC the probability of belonging to seven possible classes, which are: “brain”, “muscle”, “eye”, and “heart” for physiological sources; “line noise” and “channel noise” for extra-physiological sources; and “other” for unrecognized sources. After labeling, ICs were flagged as artifacts and removed from the EEG data if one of the following criteria was met: (1) the probability of belonging to the class “brain” was lower than 10%; (2) the probability of belonging to any other class was higher than 85%. Preprocessed EEG signals were then epoched. EEG epochs lasting 30 s were recursively extracted every 5 s up to the end of the signal. Within each EEG epoch, bad channel detection was achieved by computing four times the standard deviation of the signal within consecutive 3 s segments. If such value was greater than 100 µV in more than three segments, the corresponding EEG channel was rejected from the 30 s epoch.

Within each EEG epoch, brain rhythms were extracted using a 6th-order bidirectional Butterworth filter considering the following frequency bands: delta (1–4 Hz), theta (4–8 Hz), alpha (8–12 Hz), sensorimotor rhythm (SMR, 12–15 Hz), beta (15–30 Hz), and gamma (>30 Hz). Then, the PSD of each brain rhythm was computed using the Welch’s overlapped segment averaging estimator (4 s segments with 50% overlap) and quantified in terms of area under the curve using the trapezoidal method. Such estimation of rhythm power will be referred to as power spectral energy. Eventually, 37 engagement indexes were computed. These ratio indexes are defined as the ratio of the power spectral energy of two or more brain rhythms, and their mathematical formulation is reported in Table 1 [9].

### 3.3. Statistics

**Figure 2:**
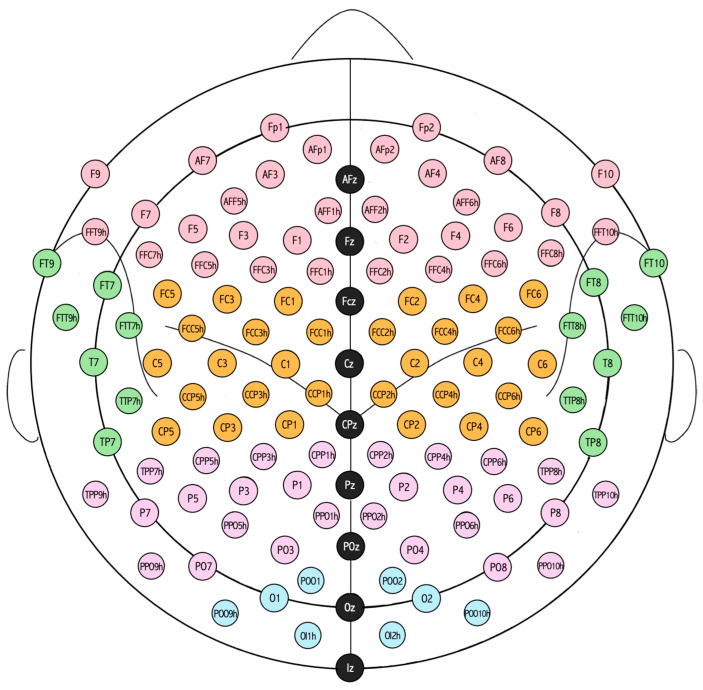
Arrangement and spatial distribution of the 120 EEG electrodes into five brain regions. Pink electrodes correspond to the frontal region, orange electrodes correspond to the central region, green electrodes correspond to the temporal region, purple electrodes correspond to the parietal region, and light blue electrodes correspond to the occipital region. Black electrodes, which represent the midline used to divide each region into left and right sides, were not considered.

EEG channels were grouped in five brain regions, as shown in Figure 2. The brain regions considered were the frontal, the central, the temporal, the parietal and the occipital. While the left and right sides of the temporal region are distant, the sides of the other regions are adjacent, and all are defined relative to the sagittal midline. For each EEG epoch, engagement indexes were averaged (mean) over channels belonging to the same brain region, distinguishing between the left and right sides of each region. Thus, since EEG epochs were recursively extracted every 5 s, a trend of each feature (i.e., engagement index) was obtained per subject and per brain region side. The median trend of each engagement index over all subjects was then computed within each brain region side and smoothed using a moving average filter. The median computation was preceded by the synchronization of the three phases of the experimental protocol (i.e., resting phase, game phase and recovery phase), taking the beginning of the phase as the reference time instant. In addition, considering only EEG epochs that pertain to only one phase of the protocol (i.e., excluding the transition epochs, which simultaneously cover different phases), the engagement index trends were discretized by computing the median value of each feature every 2.5 min, yielding 2 values in the resting phase, 31 values in the game phase and 3 values in the recovery phase, per subject. All paired comparisons between consecutive values were performed using the Wilcoxon signed-rank test, setting the statistical significance (*p*) to 0.05, resulting in a total of 35 comparisons.

To account for inter-individual variability and to assess potential interaction effects between brain region and phase of play, linear mixed-effects models were fitted for each index. Phase (rest, game, recovery) and brain region were included as fixed effects, along with their interaction (Phase × Region), while subject was modeled as a random intercept. Prior to model fitting, index values were log-transformed. For indexes showing a significant Phase × Region interaction, post hoc pairwise phase contrasts were evaluated separately within each brain region using the corresponding fixed-effect estimates.

Then, to investigate condition-related differences during gameplay, a second set of linear mixed-effects models was fitted by restricting the analysis to the game phase. Condition of play (ball machine or human player) and brain region were included as fixed effects, along with their interaction (Condition × Region), while the subject was modeled as a random intercept. Index values were log-transformed prior to model fitting. For indexes showing a significant Condition × Region interaction, post hoc pairwise condition contrasts were evaluated separately within each brain region using model-based fixed-effect estimates. In both analyses, statistical significance of fixed effects was assessed using mixed-effects ANOVA, and *p*-values were corrected for multiple comparisons across indexes using the Benjamini–Hochberg false discovery rate (FDR) procedure.

## 4. Results

**Figure 3:**
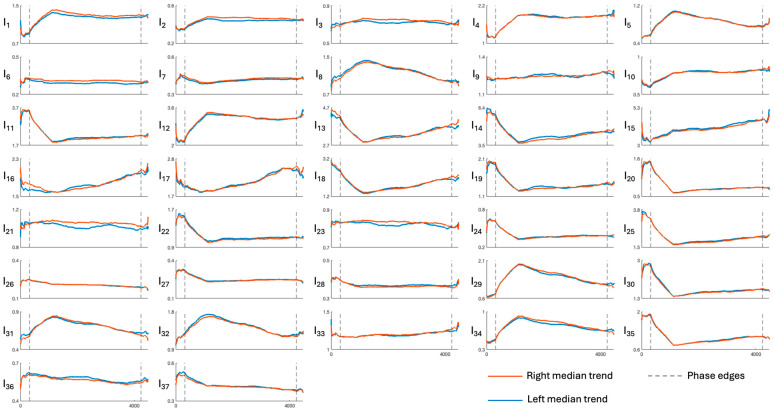
Median trends of engagement indexes in the frontal region (limits of x-axes are reported only on the bottom panels of each column, referring to the entire column). The figure is divided into 37 panels, one per engagement index. Each panel shows the median, with orange and blue representing the right and left sides of the region, respectively. Dotted lines outline the protocol phases. Indexes I_1_–I_4_ are the most frequently used in the literature; for further details see Table 1.

**Figure 4:**
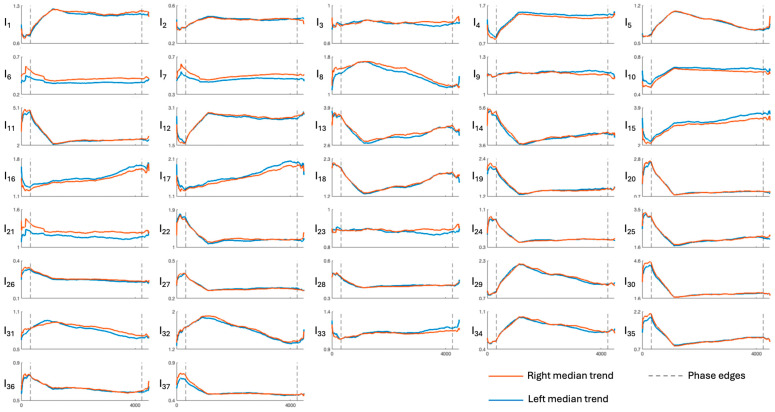
Median trends of engagement indexes in the central region (limits of x-axes are reported only on the bottom panels of each column, referring to the entire column). The figure is divided into 37 panels, one per engagement index. Each panel shows the median, with orange and blue representing the right and left sides of the region, respectively. Dotted lines outline the protocol phases. Indexes I_1_–I_4_ are the most frequently used in the literature; for further details see Table 1.

**Figure 5:**
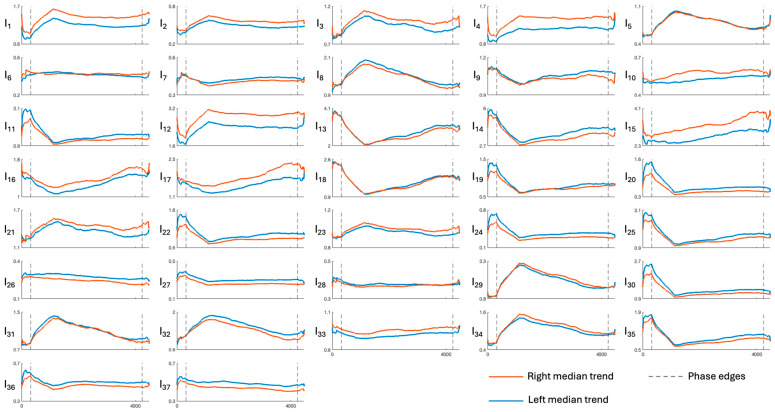
Median trends of engagement indexes in the temporal region (limits of x-axes are reported only on the bottom panels of each column, referring to the entire column). The figure is divided into 37 panels, one per engagement index. Each panel shows the median, with orange and blue representing the right and left sides of the region, respectively. Dotted lines outline the protocol phases. Indexes I_1_–I_4_ are the most frequently used in the literature; for further details see Table 1.

**Figure 6:**
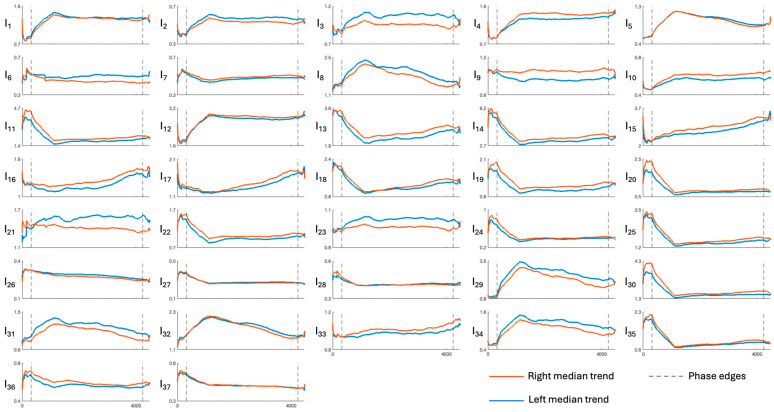
Median trends of engagement indexes in the parietal region (limits of x-axes are reported only on the bottom panels of each column, referring to the entire column). The figure is divided into 37 panels, one per engagement index. Each panel shows the median, with orange and blue representing the right and left sides of the region, respectively. Dotted lines outline the protocol phases. Indexes I_1_–I_4_ are the most frequently used in the literature; for further details see Table 1.

**Figure 7:**
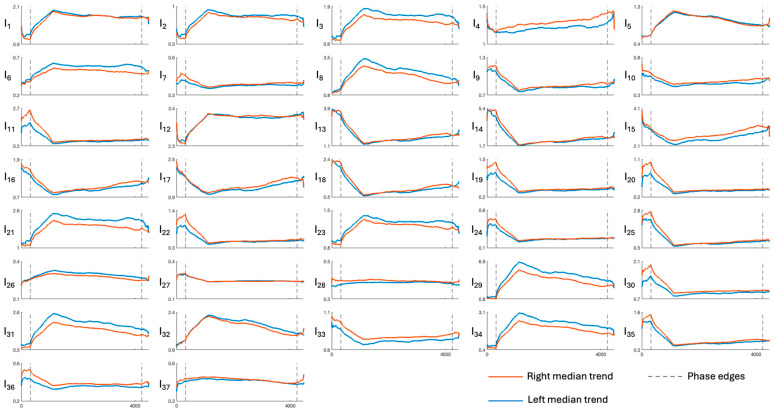
Median trends of engagement indexes in the occipital region (limits of x-axes are reported only on the bottom panels of each column, referring to the entire column). The figure is divided into 37 panels, one per engagement index. Each panel shows the median, with orange and blue representing the right and left sides of the region, respectively. Dotted lines outline the protocol phases. Indexes I_1_–I_4_ are the most frequently used in the literature; for further details see Table 1.

Figure 3, Figure 4, Figure 5, Figure 6 and Figure 7 show the median temporal trends of the 37 engagement indexes computed per brain region. Each figure contains 37 panels, one per engagement index, where the trend computed in the left side of the region is depicted by a blue thick line, while the one computed in the right side of the region is depicted by an orange thick line. The vertical dashed lines, instead, outline the three phases of the experimental protocol (i.e., resting, game, and recovery), with the duration of the game phase equal to the median duration over subjects. Across all brain regions, engagement indexes exhibited changes in their temporal trends, with variations occurring primarily at the transition between the resting and the game phase, and a return toward resting-phase values during the recovery phase for some indexes. The left and right median trends largely overlap for most engagement indexes, indicating similar temporal behavior between hemispheres.

**Figure 8:**
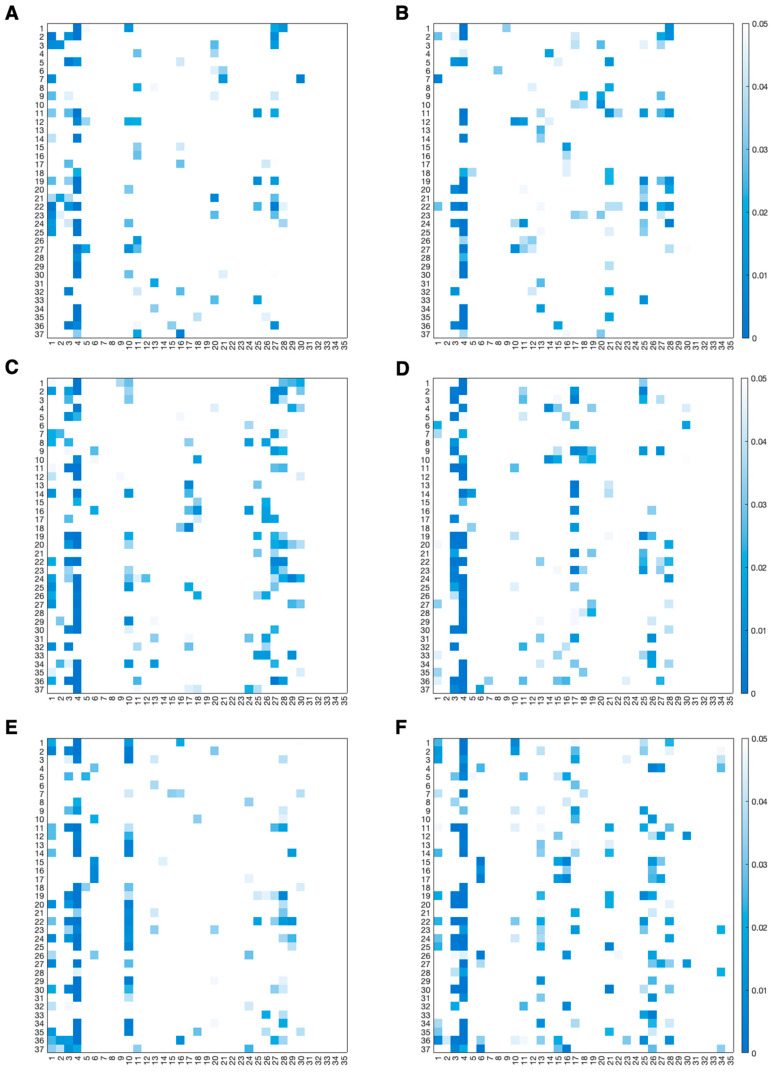
Heatmaps showing statistically significant *p*-values. Panels (**A**,**B**) refer to the frontal region; panels (**C**,**D**) refer to the central region; panels (**E**,**F**) refer to the temporal region. Left side of each region is represented in left panels (**A**,**C**,**E**); right side of each region is represented in right panels (**B**,**D**,**F**). Indexes I_1_–I_4_ are the most frequently used in the literature; for further details see Table 1.

**Figure 9:**
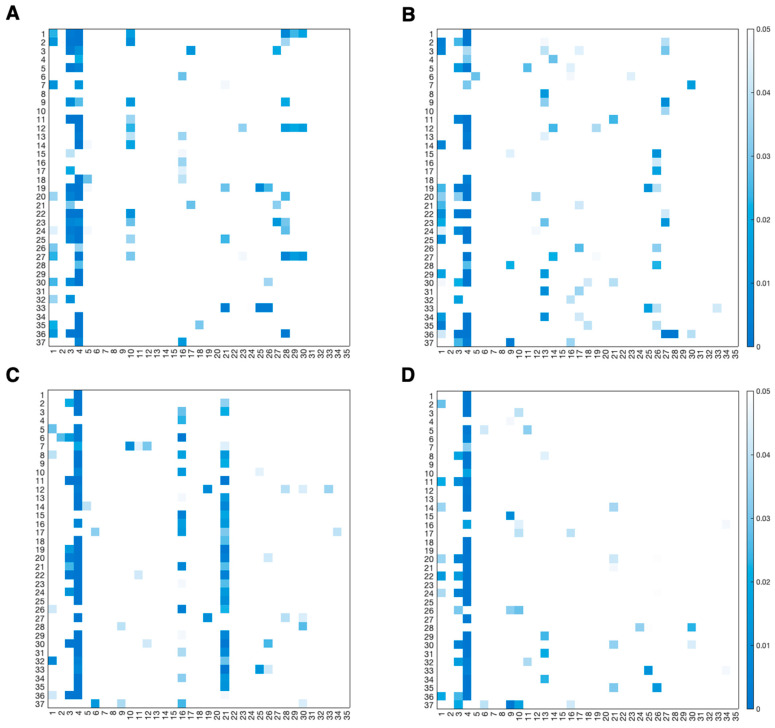
Heatmaps showing statistically significant *p*-values. Panels (**A**,**B**) refer to the parietal region, panels (**C**,**D**) refer to the occipital region. Left side of each region is represented in left panels (**A**,**C**) and right side of each region is represented in right panels (**B**,**D**). Indexes I_1_–I_4_ are the most frequently used in the literature; for further details see Table 1.

Results of the Wilcoxon signed-rank test applied to consecutive values are reported in Figure 8 and Figure 9 using heatmaps that display only statistically significant *p*-values. In each heatmap, rows correspond to engagement indexes, while columns correspond to the consecutive comparisons. More specifically, the first column represents the comparison between the epochs of the resting phase, the second column represents the comparison between the last epoch of the resting phase and the first epochs of the game phase, subsequent columns (from the 3rd to the 32nd) correspond to comparisons between consecutive epochs within the game phase, the 33rd column represents the comparison between the last epochs of the game phase and the first epochs of the recovery phase, and the last two columns represent the comparisons between the epochs of the recovery phase. Colors encode *p*-values in the range of 0–0.05, while non-significant comparisons (*p* > 0.05) correspond to cells left uncolored.

Results of the linear mixed-effects model statistics are reported in Table 2, Table 3 and Table 4. Specifically, Table 2 lists the engagement indexes exhibiting a significant main effect of phase in the linear mixed-effects ANOVA. For each index, the table reports the F-statistic and the FDR-corrected *p*-value for the phase effect. Fixed-effect estimates (β) and 95% confidence intervals (CIs) for the rest vs. game and rest vs. recovery contrasts, derived from the corresponding linear mixed-effects models, are also reported. Table 3 shows the indexes exhibiting a significant interaction between phase and region in the linear mixed-effects ANOVA. For each index, the table reports the F-statistic and the FDR-corrected *p*-value for the interaction term. Additionally, for each phase contrast (rest vs. game and rest vs. recovery), the brain regions showing significant effects are listed, together with the sign of the corresponding fixed-effect estimate (β), indicating whether the index increased or decreased in that region. Table 4 reports the results of linear mixed-effects models obtained comparing gameplay conditions (ball machine vs. human player) during the game phase. For each index with a significant condition effect, the table reports the F-statistic and FDR-corrected *p*-value from the ANOVA. Fixed-effect estimates (β) and 95% CIs are provided to quantify the differences between conditions. No index shows a significant Condition x Region interaction.

## 5. Discussion

This study aimed to characterize the psycho-emotional state induced by open-skill sport activity by exploiting EEG-derived engagement indexes and analyzing their temporal evolution. The rationale behind this work lies in the fact that the unpredictable and socially interactive nature of situations occurring in open-skill sports, combined with their emotional and cognitive demands, makes monitoring of psycho-emotional responses especially important, with objective monitoring through EEG analysis being the preferred approach.

To reach this aim, the database published by Studnicki and colleagues was exploited, as it consists of EEG data acquired before, during and after playing table tennis in a real-world scenario. The experimental protocol underlying this dataset is rooted in the principles of Mobile Brain/Body Imaging (MoBI), a new approach that enables moving beyond static laboratory settings and following the dynamic changes in cognitive demands associated with visuo-motor integration and rapid decision-making [30].

Moreover, the structured transition from baseline to active gameplay (including both ball machine and human player trials), and finally to recovery, allows for investigating baseline neural activity and recovery-related dynamics, as well as for tracking physical fatigue and cognitive engagement over time. We assessed these cognitive states using 37 EEG-derived engagement indexes. It should be noted that the literature does not always provide a unified interpretation of these measures. Indeed, studies such as ours serve a dual purpose: beyond analysis, they also contribute to the ongoing exploration and clarification of these indexes, which are particularly relevant for clinical and clinically oriented applications. Accordingly, the physiological interpretations adopted here and reported in Table 1, while grounded in previous research, should be regarded as hypotheses that are further examined and supported within the framework of our investigation.

Prior to the extraction and analysis of engagement indexes, EEG signals were filtered using the EEGLAB default function. Specifically, a 43-point zero-phase non-causal FIR filter was used. The non-causal implementation allows for correcting for group delay and preserving the temporal alignment of the signal, while the FIR design guarantees inherent stability. Powerline noise removal was achieved using CleanLine, an EEGLAB plug-in that applies a multi-taper regression to attenuate electrical noise, whose validity is reinforced by its inclusion in established EEG preprocessing pipelines, such as the PREP pipeline [35]. Then, ICA was performed using the Extended Infomax algorithm. No dimensionality reduction via principal component analysis was applied because, with principal component analysis being a variance-based method, it may discard low-variance but physiologically meaningful sources. Moreover, in EEG analysis, full-rank ICA is generally preferred, and additional rank reduction is avoided unless clearly justified. In our case, although average referencing inherently reduces data rank, no further dimensionality reduction was introduced prior to ICA. The Extended Infomax algorithm is widely validated and commonly used in EEG, as it guarantees a robust separation of both neural and artefactual source and should perform better with maximum-rank data. Artifact identification and removal was achieved using ICLabel [32], a peer-reviewed tool for EEG independent component classification validated in the field and widely adopted in EEG preprocessing pipelines [36,37,38]. The decision to adopt this automatic tool was motivated by the intention to provide a systematic and objective procedure suitable for future investigations involving larger datasets. While ICs can be identified and categorized through manual inspection, this process is time-consuming and requires expertise and training. In contrast, automated IC classification methods, such as the one employed in the present study, although potentially subject to classification inaccuracies, enable reproducible and unbiased component labeling and substantially accelerate the detection of artifactual components, particularly in studies with large sample sizes. After preprocessing, overlapping 30 s epochs were recursively extracted every 5 s. These implementation choices were motivated by the need to balance temporal resolution with signal reliability, ensuring that each segment was sufficiently long to provide stable and robust EEG-derived measurements, while still allowing for the extraction of an almost continuous temporal trend [39,40]. Given the sensitivity of EEG recordings to motion artifacts, a self-implemented algorithm for bad channel detection was also used within each 30 s epoch, whose workflow was based on signal standard deviation to capture residual local artifacts that may have persisted after ICA. This proposed analysis approach contributes to providing a structured and effective pipeline for EEG signal optimization, improving data quality and reliability. By exploiting overlapping epochs and obtaining a time trend of the observed features, our approach allows for determining whether engagement indexes are capable of tracking the transitions between different cognitive demands. In addition, although the acquisitions in the database analyzed were made with a laboratory-based benchtop EEG system, the implemented preprocessing and processing pipeline addresses the critical issues typical of acquisitions made with wearable sensors, such as noise, motion artifacts, and signal variability.

Figure 3, Figure 4, Figure 5, Figure 6 and Figure 7 show how the engagement indexes change over time in the various brain regions considered, reflecting the underlying changes of EEG rhythms. According to the level of cognitive engagement elicited by the different phases of the sport activity, the index trend could be increasing, decreasing, presenting a peak, or even flat. A primary observation is that the most pronounced variations in engagement indexes occur at the transition from the resting phase to the game phase. This suggests that the onset of the open-skill activity induces a modulation of EEG-derived engagement measures, reflecting the shift from a baseline condition to an active, cognitively and emotionally demanding mental state. Notably, indexes with beta and/or gamma in the denominator in their mathematical formulation (I_11_, I_13_, I_14_, I_19_, I_20_, I_22_, I_24_, I_25_, I_30_, I_35_) exhibit a decreasing trend in the transition from rest to game, while those with beta/alpha in their mathematical formulation (I_1_, I_2_, I_12_) exhibit an increasing trend, which is consistent with enhanced cognitive activation [5,6]. In addition, indexes I_5_, I_8_, I_29_, I_31_, I_32_, I_34_, all with delta rhythm in the denominator in their mathematical formulation, tend to increase during the game phase and subsequently return, when passing towards the recovery phase, to values comparable to those observed during the resting phase. This behavior could be attributable to an increase in delta power during recovery, as higher delta powers have been linked to reduced alertness [6,7]. The remaining indexes, instead, stay at levels different from baseline during the recovery phase, suggesting that the psycho-emotional state induced by open-skill activity does not uniformly resolve immediately after task completion, and that longer recovery intervals may be required to completely restore baseline neural states. Statistical analyses confirmed the outcomes provided by the median trend analysis. Indeed, linear mixed-effects modeling revealed significant phase-related effects for several engagement indexes, with most indexes being statistically different between the resting and the game phase, no indexes exhibiting significant changes from the game to the recovery phase, and few indexes exhibiting non-significant changes between the resting and the recovery phase. Some significant changes were also observed within the game phase; specifically, 16 out of 37 engagement indexes exhibited significant differences between playing against a ball machine and playing against a human opponent, indicating distinct patterns of cortical engagement associated with the two conditions. Similar analyses focusing on the 15 min blocks within the game phase could further elucidate the mechanisms underlying these within-game variations and investigate fatigue-related effects, which may accumulate over time based on the protocol design.

Most indexes show consistent temporal trends across the various brain regions considered, with the temporal and central regions being those with the greatest number of significant changes in index trends, and the occipital region the one with the fewest. Moreover, some indexes displayed different patterns in the occipital region. Specifically, indexes with alpha in the denominator that show an increasing trend in all other regions show a decreasing trend in the occipital one. This may be due to increased alpha power, which reflects the inhibition-timing hypothesis, according to which brain regions not directly involved in the task are suppressed in favor of task-related ones [12,41]. Similarly, indexes with theta in the denominator increased in both the occipital and temporal regions, rather than decreasing as in the other brain regions, possibly suggesting decreased theta power. Within each brain region, instead, the spatial analysis reveals a high degree of similarity between the left and right hemispheres, hinting that the psycho-emotional processes involved in the task are not lateralized or confined to specific cortical areas.

Some limitations of this study should be acknowledged, pointing out the possible evolution of the study. First, although we employed linear mixed-effects models to account for inter-subject variability, the quite small population size may limit generalizability. It is important to note that this research represents a secondary analysis of the ‘Real World Table Tennis’ database by Studnicki et al., and the cohort size was predetermined by the available open-source data. While larger samples are always preferable for statistical robustness, publicly available EEG repositories recorded during open-skill sports are currently rare. Second, the participant pool lacks explicit definition of diversity in skill levels. Future works could expand the sample size, explore different types of open-skill activities, and investigate the different contexts of training and competition, while also examining the influence of task difficulty or expertise level on engagement dynamics. Comparing amateur and professional athletes would add an interesting perspective, particularly for assessing psycho-emotional involvement in relation to experience and psychomotor skills acquired and consolidated at different levels of expertise. Furthermore, having physiological measures that do not interfere with participation (i.e., a method minimally invasive in sports practice and movement execution) is advantageous. Thus, wearable sensors represent a preferable solution for this kind of application. As a consequence, the implemented pipeline should be validated on EEG signals acquired by wearable technology. Furthermore, while the present study used spectral power-based engagement indexes due to their more direct physiological interpretability and low computational cost, sophisticated deep-learning architectures, such as the spatio-temporal representation fusion learning network [42] and hierarchical spatial–frequency networks [43], primarily used for emotion recognition, could be assessed in terms of their adaptability for monitoring mental engagement during sports activity, alongside an appropriate evaluation of their physiological interpretability. These future investigations could contribute to further validating and refining the proposed framework. At the same time, the main contribution of our study is that it provides useful insights for improving sports training design and supporting mental well-being, while also advancing research in the field of sports psychology and pedagogy and contributing to the ongoing investigation of the clinical role and physiological interpretation of engagement indexes in different scenarios. Psychophysiological measures like the ones addressed in the present work could also represent a means of understanding the role of stress in acquiring sporting skills and, from a broader perspective, of further confirming that practicing sport promotes positive mental health.

## 6. Conclusions

This study suggests that engagement indexes are effective EEG-derived biomarkers able to reflect changes in mental engagement induced by open-skill sport activities such as table tennis. Indeed, most indexes reliably captured the transition from the resting phase to the game phase in all cortical regions, exhibiting an increasing trend with beta/alpha in their definition, and a decreasing trend when high-frequency rhythms were in the denominator.

## Figures and Tables

**Table 1:** Mathematical formulation and physiological interpretation of the engagement indexes. Indexes I_1_–I_4_ are the most frequently used in the literature to study mental involvement [8,9,33,34].

Index	Formula	Interpretation	Index	Formula	Interpretation
**I_1_**	$\frac{\beta }{\alpha }$	mostly used as index of arousal	**I_20_**	$\frac{\alpha }{\gamma }$	used as index of attention
**I_2_**	$\frac{\beta }{\theta +\alpha }$	mostly used as index of focused attention	**I_21_**	$\frac{SMR+\beta }{\theta }$	used as index of concentration
**I_3_**	$\frac{\beta }{\theta }$	related to attentional control and mental fatigue	**I_22_**	$\frac{\theta +\alpha }{\beta +\gamma }$	introduced to assess vigilance
**I_4_**	$\frac{\theta }{\alpha }$	related to mental workload	**I_23_**	$\frac{\alpha +\beta }{\theta +\alpha }$	introduced to assess vigilance
**I_5_**	$\frac{\theta }{\delta }$	linked to working memory	**I_24_**	$\frac{\alpha }{\beta +\gamma }$	introduced to assess vigilance
**I_6_**	$\frac{SMR}{\theta }$	mostly used for neurofeedback training	**I_25_**	$\frac{\delta +\theta +\alpha }{\beta +\gamma }$	introduced to assess vigilance
**I_7_**	$\frac{SMR}{\beta }$	mostly used for neurofeedback training	**I_26_**	$\frac{\alpha }{\delta +\theta +\alpha }$	introduced to assess vigilance
**I_8_**	$\frac{\alpha +\beta }{\delta }$	used as index of mental fatigue	**I_27_**	$\frac{\alpha }{\theta +\alpha +\beta }$	introduced to assess vigilance
**I_9_**	$\frac{\theta +\alpha }{\alpha +\beta }$	used as index of mental fatigue	**I_28_**	$\frac{\beta }{\theta +\gamma }$	introduced to assess vigilance
**I_10_**	$\frac{\theta }{\alpha +\beta }$	used as index of mental fatigue	**I_29_**	$\frac{\beta +\gamma }{\delta }$	introduced to assess vigilance
**I_11_**	$\frac{\theta +\alpha }{\gamma }$	used as index of working memory and mental fatigue	**I_30_**	$\frac{\alpha +\beta }{\gamma }$	introduced to assess vigilance
**I_12_**	$\frac{\theta +\beta }{\alpha }$	used as index of cognitive load	**I_31_**	$\frac{\alpha +\gamma }{\delta +\theta }$	introduced to assess vigilance
**I_13_**	$\frac{\delta +\theta }{\beta }$	used as index of mental fatigue	**I_32_**	$\frac{\theta +\alpha }{\delta }$	introduced to assess vigilance
**I_14_**	$\frac{\delta +\theta +\alpha }{\beta }$	used as index of mental fatigue	**I_33_**	$\frac{\theta +\beta }{\alpha +\gamma }$	introduced to assess vigilance
**I_15_**	$\frac{\delta +\theta }{\alpha }$	used as index of executive load	**I_34_**	$\frac{\beta +\gamma }{\delta +\theta }$	introduced to assess vigilance
**I_16_**	$\frac{\delta +\theta }{\alpha +\beta }$	used of index of consciousness	**I_35_**	$\frac{\delta +\alpha }{\theta +\gamma }$	introduced to assess vigilance
**I_17_**	$\frac{\delta }{\alpha }$	used as index of mental stress	**I_36_**	$\frac{\theta +\alpha }{\delta +\beta +\gamma }$	introduced to assess vigilance
**I_18_**	$\frac{\delta }{\beta }$	used as index of mental stress	**I_37_**	$\frac{\alpha +\beta }{\delta +\theta +\gamma }$	introduced to assess vigilance
**I_19_**	$\frac{\theta }{\gamma }$	used to assess working memory and attention			

**Table 2:** Indexes showing a significant main effect of phase in the ANOVA test. F-statistics (degrees of freedom) and FDR-corrected *p*-values were obtained from ANOVA on linear mixed-effects models; effect sizes (β) and 95% confidence intervals (CIs) for phase contrasts were derived from the corresponding fixed-effect estimates.

Index	F-Statistics	FDR *p*-Value	Rest vs. Game		Rest vs. Recovery	
			β	CI	β	CI
I_1_	19.13 (2710)	2.5 × 10^−8^	0.18	0.11, 0.24	0.18	0.11, 0.25
I_2_	6.85 (2710)	0.001	0.11	0.04, 0.17	0.10	0.04, 0.17
I_11_	21.09 (2710)	4.3 × 10^−9^	0.04	−0.34, −0.17	0.04	−0.33, −0.15
I_12_	21.44 (2710)	3.3 × 10^−9^	0.16	0.10, 0.22	0.17	0.11, 0.23
I_19_	8.32 (2710)	4.1 × 10^−4^	−0.17	−0.26, −0.08	−0.15	−0.24, −0.06
I_20_	32.63 (2710)	1.5 × 10^−13^	−0.34	−0.43, −0.25	−0.33	−0.42, −0.23
I_22_	14.59(2710)	1.3 × 10^−6^	−0.17	−0.25, −0.10	−0.17	−0.24, −0.10
I_24_	27.15 (2710)	2.1 × 10^−11^	−0.25	−0.33, −0.18	−0.25	−0.33, −0.17
I_27_	21.83 (2710)	2.4 × 10^−9^	−0.10	−0.14, −0.06	−0.11	−0.14, −0.07
I_30_	33.71 (2710)	6.3 × 10^−14^	−0.26	−0.33, −0.19	−0.25	−0.32, −0.18

**Table 3:** Indexes showing a significant Phase x Region interaction in the ANOVA test. F-statistics (degrees of freedom) and FDR-corrected *p*-values were obtained from ANOVA on linear mixed-effects models; regions (β sign) showing significant effects after post hoc testing are listed.

Index	F-Statistics	FDR *p*-Value	Significant Regions (Effect Size)	
			Rest vs. Game	Rest vs. Recovery
I_3_	3.44 (18,710)	4.1 × 10^−6^	OL, OR (β > 0)	OL, OR (β > 0)
I_4_	3.57 (18,710)	1.9 × 10^−5^	FL, FR, CL, CR, TL, TR, PL, PR (β > 0)	FL, FR, CL, CR, TL, TR, PL, PR (β > 0)
I_5_	3.11 (18,710)	2.9 × 10^−7^	all regions (β > 0)	all regions (β > 0)
I_7_	3.99 (18,710)	1.7 × 10^−7^	OL, OR (β > 0)	OL, OR (β > 0)
I_8_	4.53 (18,710)	6.1 × 10^−9^	FL, FR, TL, TR, PL, PR, OL, OR (β > 0)	FL, FR, PL, PR, OL, OR (β > 0)
I_9_	3.86 (18,710)	3.5 × 10^−7^	OL, OR (β < 0)	OL, OR (β < 0)
I_10_	4.84 (18,710)	9.4 × 10^−10^	FL, FR, CL, CR, PL, PR (β > 0) OL, OR (β < 0)	FL, FR, CL, CR, PL, PR (β > 0) OL, OR (β < 0)
I_13_	3.31 (18,710)	9.2 × 10^−6^	FL, FR, TL, TR, PL, PR, OL, OR (β < 0)	FL, FR, PL, PR, OL, OR (β < 0)
I_14_	2.26 (18,710)	0.003	all regions (β < 0)	all regions (β < 0)
I_15_	2.99 (18,710)	5.6 × 10^−5^	CL, CR (β > 0) OL, OR (β < 0)	CL, CR (β > 0) OL, OR (β < 0)
I_16_	3.93 (18,710)	2.5 × 10^−7^	OL, OR (β < 0)	OL, OR (β < 0)
I_17_	2.83 (18,710)	1.3 × 10^−4^	OL, OR (β < 0)	OL, OR (β < 0)
I_18_	3.25 (18,710)	1.3 × 10^−5^	all regions (β < 0)	FL, FR, PL, PR, OL, OR (β < 0)
I_21_	3.76 (18,710)	6.2 × 10^−7^	OL, OR (β > 0)	OL, OR (β > 0)
I_23_	3.82 (18,710)	4.6 × 10^−7^	OL, OR (β > 0)	OL, OR (β > 0)
I_25_	1.73 (18,710)	0.04	all regions (β < 0)	all regions (β < 0)
I_26_	3.17 (18,710)	2.1 × 10^−5^	CL, CR (β < 0) OL, OR (β > 0)	CL, CR (β < 0) OL, OR (β > 0)
I_28_	2.94(18,710)	7.3 × 10^−5^	FL, FR, CL, CR, TL, TR, PL, PR (β < 0)	FL, FR, CL, CR, TR, PL, PR (β < 0)
I_29_	3.14 (18,710)	2.5 × 10^−5^	all regions (β > 0)	all regions (β > 0)
I_31_	3.99 (18,710)	1.7 × 10^−7^	FL, FR, TL, TR, PL, PR, OL, OR (β > 0)	FL, FR, PL, PR, OL, OR (β > 0)
I_32_	2.77 (18,710)	5.9 × 10^−7^	FL, FR, TL, TR, PL, PR, OL, OR (β > 0)	FL, FR, PL, PR, OL, OR (β > 0)
I_33_	2.83 (18,710)	1.3 × 10^−4^	OL, OR (β < 0)	OL, OR (β < 0)
I_34_	2.91 (18,710)	8.5 × 10^−5^	all regions (β > 0)	all regions (β > 0)
I_35_	1.72 (18,710)	0.04	all regions (β < 0)	all regions (β < 0)
I_37_	3.61 (18,710)	1.5 × 10^−6^	CL, CR, PL, PR (β < 0) OL, OR (β > 0)	CL, CR, PL, PR (β < 0)

FL: frontal left; FR: frontal right; CL: central left; CR: central right; TL: temporal left; TR: temporal right; PL: parietal left; PR: parietal right; OL: occipital left; OR: occipital right.

**Table 4:** Indexes showing a significant main effect of game condition in the ANOVA test. F-statistics (degrees of freedom) and FDR-corrected *p*-values were obtained from ANOVA on linear mixed-effects models; effect sizes (β) and 95% confidence intervals (CIs) for phase contrasts were derived from the corresponding fixed-effect estimates.

Index	F-Statistics	FDR *p*-Value	Ball Machine vs. Human Player	
			β	CI
I_5_	19.13 (1480)	8.2 × 10^−4^	−0.05	−0.08, −0.02
I_8_	7.72 (1480)	0.01	−0.08	−0.15, −0.03
I_13_	5.68 (1480)	0.04	0.07	0.01, 0.12
I_14_	5.78 (1480)	0.04	0.06	0.01, 0.11
I_16_	5. 30(1480)	0.03	0.06	0.01, 0.11
I_17_	6.01 (1480)	0.04	0.07	0.01, 0.13
I_18_	7.47 (1480)	0.03	0.09	0.02, 0.15
I_19_	6.44 (1480)	0.01	0.09	0.02, 0.15
I_25_	7.95 (1480)	0.02	0.08	0.02, 0.13
I_26_	5.60 (1480)	0.04	−0.04	−0.07, −0.01
I_29_	9.65 (1480)	5.5 × 10^−3^	−0.12	−0.19, −0.04
I_31_	7.01 (1480)	0.02	−0.08	−0.14, −0.02
I_32_	9.29 (1480)	0.01	−0.06	−0.10, −0.02
I_33_	6.60 (1480)	0.02	0.04	0.01, 0.07
I_34_	7.51 (1480)	0.01	−0.09	−0.16, −0.02
I_35_	9.80 (1480)	0.01	0.06	0.02, 0.10

## Data Availability

The data used in this study are freely available on OpenNeuro (https://doi.org/10.18112/openneuro.ds004505.v1.0.4, accessed on 28 April 2025).

## Abbreviations

The following abbreviations are used in this manuscript:

MoBI	Mobile Brain/Body Imaging
CI	Confidence Interval
EEG	Electroencephalography
FDR	False Discovery Rate
FFT	Fast Fourier Transform
FIR	Finite Impulse Response
IC	Independent Component
ICA	Independent Component Analysis
IMU	Inertial Measurement Unit
PSD	Power Spectral Density
SMR	Sensorimotor Rhythm
